# Speed of thermal adaptation of terrestrial vegetation alters Earth’s long-term climate

**DOI:** 10.1126/sciadv.adj4408

**Published:** 2024-03-01

**Authors:** Julian Rogger, Benjamin J. W. Mills, Taras V. Gerya, Loïc Pellissier

**Affiliations:** ^1^ Swiss Federal Institute of Technology Zurich, Department of Earth Sciences, Zurich, Switzerland.; ^2^ Swiss Federal Institute of Technology Zurich, Department of Environmental Systems Science, Zurich, Switzerland.; ^3^ University of Leeds, School of Earth and Environment, Leeds, UK.; ^4^ Swiss Federal Institute for Forest, Snow and Landscape Research, Birmensdorf, Switzerland.

## Abstract

Earth’s long-term climate is driven by the cycling of carbon between geologic reservoirs and the atmosphere-ocean system. Our understanding of carbon-climate regulation remains incomplete, with large discrepancies remaining between biogeochemical model predictions and the geologic record. Here, we evaluate the importance of the continuous biological climate adaptation of vegetation as a regulation mechanism in the geologic carbon cycle since the establishment of forest ecosystems. Using a model, we show that the vegetation’s speed of adaptation to temperature changes through eco-evolutionary processes can strongly influence global rates of organic carbon burial and silicate weathering. Considering a limited thermal adaptation capacity of the vegetation results in a closer balance of reconstructed carbon fluxes into and out of the atmosphere-ocean system, which is a prerequisite to maintain habitable conditions on Earth’s surface on a multimillion-year timescale. We conclude that the long-term carbon-climate system is more sensitive to biological dynamics than previously expected, which may help to explain large shifts in Phanerozoic climate.

## Teaser

The balance between biological adaptation and past environmental changes has shaped Earth’s climate evolution over deep time.

## INTRODUCTION

**Fig. 1.**
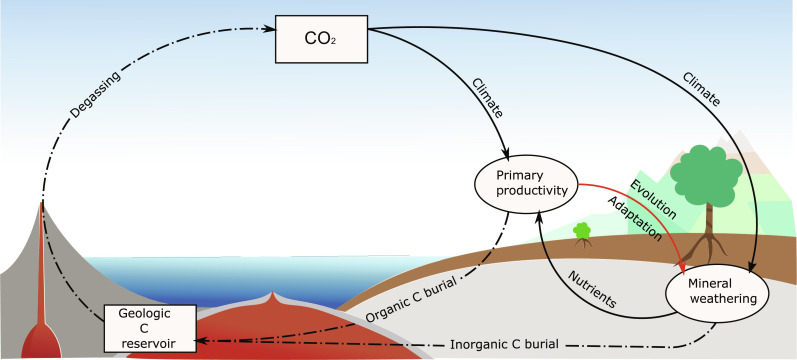
Representation of long-term global carbon cycle. Squares indicate carbon pools; discontinuous arrows indicate carbon fluxes; round symbols represent key regulation mechanisms of the atmosphere-ocean carbon mass balance, with influential variables given by continuous arrows. The red arrow depicts the studied influence of a spatially explicit and thermally adapting vegetation on rates of primary productivity and mineral weathering enhancement and, thus, long-term organic and inorganic carbon burial.

Earth’s long-term climate is controlled by the balance of CO_2_ released from geological reservoirs during volcanism, metamorphism, and the oxidation of buried organic material and the consumption of atmospheric CO_2_ through the weathering of silicate rocks as well as the burial of organic carbon (*1*). The terrestrial vegetation affects the global carbon cycle in two major ways (Fig. 1): First, primary productivity determines the amount of photosynthetically fixed atmospheric CO_2_ and contributes to the burial of carbon in organic forms. Second, plants and their symbiotic associations (e.g., mycorrhiza) enhance the CO_2_ consumption by silicate mineral weathering through multiple feedback mechanisms. These include the release of reactive species into the soil volume (e.g., hydrogen ions, organic acids, and chelators) to acquire growth-limiting nutrients, increasing the soil CO_2_ concentrations during respiration, increasing the concentration of reactive species in the soil following plant litter decomposition, and, finally, by mediating physical processes such as an intensified evapotranspiration-precipitation cycle or the stabilization of soils, permitting continued chemical weathering of primary minerals (*2*–*4*). Together, these processes result in vegetated areas showing up to 10-fold increases in weathering rates compared to unvegetated land (*5*). The strength of the weathering enhancement increases with high rates of primary productivity and plant biomass, driven by increased carbon, nutrient, and water fluxes (fig. S1) (*3*, *5*–*9*). Major transitions in Earth’s climate and atmospheric composition are being attributed to the rise and evolution of terrestrial plants and subsequent changes in carbon burial rates and silicate weathering (*2*, *10*–*13*). However, state-of-the-art numerical models of the long-term carbon cycle are poorly equipped to study the effects of vegetation dynamics and its evolution on Earth’s climate, which is considered a key reason for remaining discrepancies between numerical model predictions of CO_2_ and temperature compared to paleoclimate proxies (*14*).

Biogeochemical models of the Phanerozoic Earth tend not to consider ecological or evolutionary changes in the terrestrial vegetation beyond certain major events such as the rise of deep rooting vascular trees between 350 and 400 Ma before present (*14*–*16*). This neglects the fact that the vegetation and its interaction with biogeochemical cycles is subject to continuous change, driven by an ever-changing environment, including altered climate, landscapes, and continental configurations to which organisms adapt through ecological (e.g., movement and competition) and evolutionary (e.g., adaptive evolution) processes (*17*, *18*). For example, plant thermal tolerances and the degree of adaptation to the prevailing climatic conditions determine the potential and geographical distribution of primary productivity (*19*, *20*). Accordingly, climatic changes exert a strong selective pressure on ecosystems, and in cases where they exceed the vegetation’s eco-evolutionary adaptation capacity, this will result in a fitness and productivity reduction (*18*, *21*–*24*). The balance between the rate of environmental change and the speed of biological adaptation is thus expected to affect the carbon-climate regulation system by controlling rates of primary productivity and the strength of the vegetation-silicate weathering feedback.

On geological timescales, carbon fluxes to and from geological reservoirs are large compared to the amount of carbon that can be stored in the combined pools of the atmosphere, oceans, and biosphere. As a result, small imbalances of less than 5% between CO_2_ release and removal fluxes would result in extreme thermal conditions (i.e., extreme hothouse or complete depletion of atmospheric CO_2_) within a few million years and would cause all life to perish (*25*, *26*). Commonly referred to as the paleothermostat mechanism, atmospheric CO_2_ is thus assumed to stabilize within a few hundred thousand years (<500 ka) at a concentration at which climatic conditions result in a close balance of carbon removal and release fluxes (*25*–*28*). This has been shown to be the case for the late Pleistocene and the longer-term Cenozoic carbon fluxes estimated from geochemical proxies (maximum imbalance between carbon release and removal fluxes of 1 to 2% for late Pleistocene, less than 5% for Cenozoic) (*29*, *30*). Therefore, also on a Phanerozoic timescale, an accurate process-based model of organic and inorganic carbon fluxes informed by paleoclimate and paleogeography should result in an atmosphere-ocean carbon mass balance closely fluctuating around a steady state when calculated for an atmospheric CO_2_ concentration within the range observed in the geologic proxy record.

Here, we investigate the impact of thermal adaptation of the terrestrial vegetation through ecological and evolutionary processes on global organic and inorganic carbon fluxes by reconstructing the atmosphere-ocean carbon mass balance over the last 390 Ma, i.e., since the evolution of forest ecosystems. The process and speed of biotic adaptation to changing climatic conditions is found to be of key importance for obtaining a close balance of reconstructed carbon release and removal fluxes. Our results emphasize the role of continuous evolutionary and ecological processes in regulating the global carbon cycle and Earth’s climate over geological time.

### Reconstruction of carbon fluxes and eco-evolutionary trajectories since the mid-Paleozoic

For the estimation of carbon fluxes, we compiled a spatially resolved paleoenvironmental reconstruction using topographic reconstructions (*31*), proxy data on atmospheric CO_2_ and O_2_ concentrations (*32*, *33*), as well as an intermediate complexity climate model to obtain climatic fields of temperature, aridity, runoff, and radiation (*34*). We limit the reconstruction to the last 390 Ma, which we take as the approximate time of the emergence and spreading of deep rooting vascular plants (*16*). Before this time, we may expect a change in vegetation dynamics not captured by the model framework (e.g., different physiology and restricted geographical range). To this proxy-based climatic and topographic reconstruction, we apply carbon flux equations from well-known numerical deep time biogeochemical models (see Materials and Methods) to approximate the major carbon release and removal fluxes to and from the atmosphere-ocean system at 500-ka time intervals. These include the weathering of silicate minerals on land, as a function of erosion, runoff, temperature, as well as biotic weathering enhancement (maximum 10-fold increase in highly productive areas), seafloor weathering, dependent on temperature and scaling with degassing rates, the oxidative weathering of buried organic carbon as a function of atmospheric oxygen concentrations and surface erosion, global organic and inorganic carbon degassing, taken from different reconstruction methods, marine organic carbon production as a function of sea surface temperatures, and, finally, terrestrial organic carbon production and burial as a function of radiation, aridity, temperatures, and the state of adaptation of the terrestrial vegetation to the prevailing climate.

To estimate the state of adaptation of the terrestrial vegetation to the environment, we use a spatially explicit, eco-evolutionary model “gen3sis” (*35*) that we configured to represent floras (all plant life and symbiotic organisms living in a certain location at a certain point in time) that populate terrestrial grid cells and together compose the vegetation. The modeled floras are characterized and differentiated only by a specific optimum temperature, describing the climatic niche in which they have their greatest potential for primary productivity and silicate weathering enhancement. Considering the paleoenvironmental reconstruction as input, the reconstructed climatic and geographic changes potentially result in a discrepancy between a flora’s optimum temperature and the actual local temperature. The gen3sis model approximates three eco-evolutionary mechanisms that work toward a re-adaptation of the flora to the new environment. These include (i) the dispersal of floras into surrounding terrestrial grid cells at a specified rate, enabling the migration into suitable habitats, (ii) the adaptive evolution of the flora’s optimum temperature toward the local environment at a specified speed, and (iii) the competition of floras that arrive in the same terrestrial grid cell, allowing only the best adapted flora (smallest discrepancy between optimum temperature and local temperature) to occupy a location. Applying different rates of adaptive evolution speeds and dispersal capacities, a set of eco-evolutionary trajectories are produced that give a spatially explicit estimate of the state of adaptation of the vegetation to a given environment, affecting local rates of primary productivity and weathering enhancement. Thus, favorable climatic and paleogeographic conditions (e.g., enabling effective migration) result in a high primary productivity and weathering potential, whereas abrupt environmental changes have the opposite effect, resulting in an ephemeral maladaptation of the vegetation when the environmental changes exceed the adaptation capacity of floras.

Taking present-day estimates of organic carbon burial and silicate weathering as a reference, different eco-evolutionary trajectories (i.e., the temporal and spatial distribution of vegetation adaptation to its environment) result in distinct carbon burial and weathering estimates over time. In contrast, we also run a model that does not consider eco-evolutionary dynamics and a potential lack of adaptation (corresponding to an immediately adapting vegetation as assumed in common deep time biogeochemical models), where primary productivity and plant-mediated weathering enhancement is solely controlled by abiotic conditions (radiation, water, temperatures).

The carbon mass balance calculations are conducted for different rates of thermal adaptation evolution and dispersal capacities of the terrestrial vegetation as well as for a wide range of reconstructed atmospheric CO_2_ concentrations and solid Earth carbon degassing rates. While many of the processes we model are taken from previous dynamical biogeochemical models, we emphasize that this model does not calculate an evolving CO_2_ concentration dynamically; instead, it calculates the implied carbon flux balance at a set of discrete points in time. A dynamic coupling of the eco-evolutionary model with an evolving carbon cycle would require substantially more computational time and is the focus of ongoing research and development.

## RESULTS

**Fig. 2.**
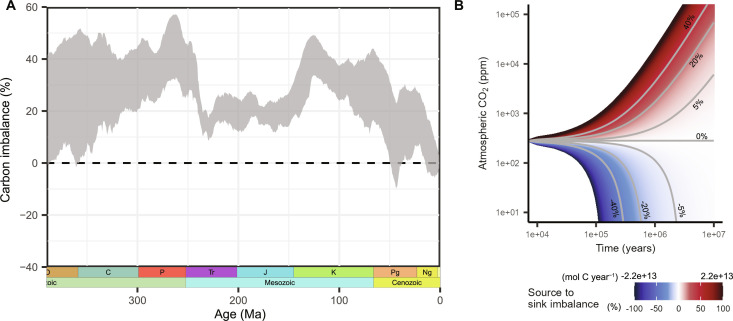
Atmosphere-ocean carbon imbalances considering a vegetation with no eco-evolutionary dynamics. (**A**) Imbalances reconstructed for the last 390 Ma in 500-ka time steps. The gray area represents the uncertainty in the mass balance for different CO_2_/climate and solid Earth degassing reconstructions. The dashed line at 0% depicts the expected mass balance according to the paleothermostat theory. (**B**) Illustration of impact of reconstructed imbalances. Colored trajectories depict the CO_2_ content that would result when a certain imbalance was maintained over time, assuming a pre-industrial atmosphere-ocean carbon content (3 × 10^18^ mol C; 280 ppm atmospheric CO_2_) and fluxes as basis (total sinks of ≈ 2.2 × 10^13^ mol C year^−1^), calculated based on the model by Kump and Arthur (*52*) for atmospheric CO_2_ (see Materials and Methods).

When reconstructing the atmosphere-ocean carbon mass balance with an immediately adapting vegetation (i.e., no eco-evolutionary dynamics and no variation in state of adaptation), a strong overall imbalance toward carbon release is estimated (average flux imbalance of *I* = 26 ± 12%) (Fig. 2A). Thus, within the explored range of climatic conditions derived from the CO_2_ proxy record (fig. S2), the calculated carbon sinks do not match the reconstructed rates of carbon release by degassing and oxidative weathering, with the surplus indicating either an underestimation of the carbon sink strength or an overestimation of reconstructed rates of carbon release. Sustained imbalances of this magnitude are implausible as they would have resulted in a runaway greenhouse effect and extreme climatic conditions on Earth within a couple of millions of years as illustrated in Fig. 2B. The reconstructed carbon surplus when not considering eco-evolutionary adaptation is consistent with the overprediction of Phanerozoic CO_2_ levels in forward biogeochemical models that do not include such biological dynamics (*14*).

**Fig. 3.**
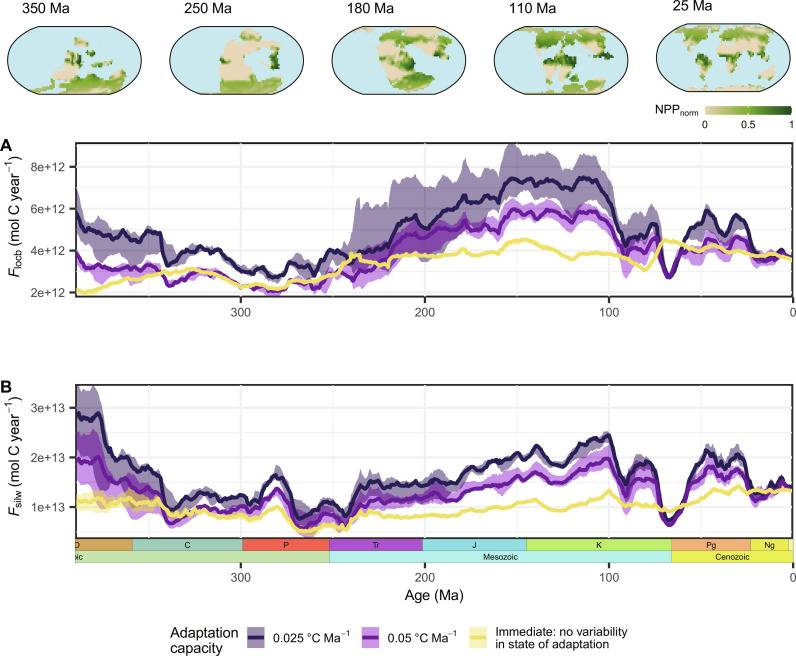
Estimated carbon fluxes for different modes of vegetation adaptation to climatic changes. (**A**) Land-derived organic carbon burial (*F*_locb_) and (**B**) silicate weathering (*F*_silw_) for two slow-adapting vegetation implementations and an immediate adaptation implementation (no eco-evolutionary dynamics). The line indicates the median, and the colored area represents the uncertainty due to different climatic reconstructions. The temporal evolution of the carbon fluxes is driven by relative changes in the vegetation’s adaptation state compared to the present day (except in the “immediate” scenario), changes in the abiotic environmental conditions, and the total area of productive land. The global plots on top depict the normalized primary productivity and weathering potential (*NPP*_norm_; see Materials and Methods) over time for a vegetation implementation with floras having a speed of in situ adaptive evolution of 0.05°C Ma^−1^ (dispersal of 1300 km Ma^−1^). The temporal evolution of the global average *NPP*_norm_ for sample scenarios is shown in fig. S3.

Considering eco-evolutionary adaptation dynamics results in an increased variability of modeled organic and inorganic carbon burial fluxes due to differences in the vegetation’s adaptation state (Fig. 3). In the eco-evolutionary model, extended periods in a favorable paleogeographic setting that enables efficient dispersal, combined with stable and favorable climatic conditions, result in an increased efficiency of the terrestrial vegetation to become adapted to local climatic conditions. The larger abundance of well-adapted floras, especially in regions of high importance for silicate weathering (high erosion, runoff, and temperatures), results in increased organic carbon production and weathering enhancement compared to periods of environmental disturbances or generally less favorable abiotic conditions for plant growth. Accordingly, highest rates of organic carbon burial and silicate weathering are modeled during the mid-Mesozoic, for which warm and stable climatic conditions are reconstructed (fig. S2). Overall, our models that include eco-evolutionary dynamics predict a larger abundance of well-adapted floras during many times in the geologic past compared to the vegetation’s adaptation state modeled for the present day, which is negatively influenced by the late Cenozoic decline in temperatures. Given that all models are calibrated to reproduce the same global reference carbon fluxes for the present day, these relative differences in adaptation result in higher estimates of carbon burial and biotic enhancement of weathering in the geologic past than have previously been considered. Lower climate adaptation capacities thereby result in a more sensitive response of the vegetation to tectonic and climatic changes and, thus, larger differences in carbon fluxes between stable and unstable environmental conditions. In contrast, less variability in carbon fluxes is observed for the immediately adapting vegetation implementation due to the assumption of an equally well-adapted vegetation throughout the model period. In this case, organic carbon production and biotic weathering enhancement are solely affected by differences in the abiotic environmental conditions and there is no possibility of higher or lower rates of carbon burial due to a higher or lower abundance of well-adapted floras than at present, respectively.

**Fig. 4.**
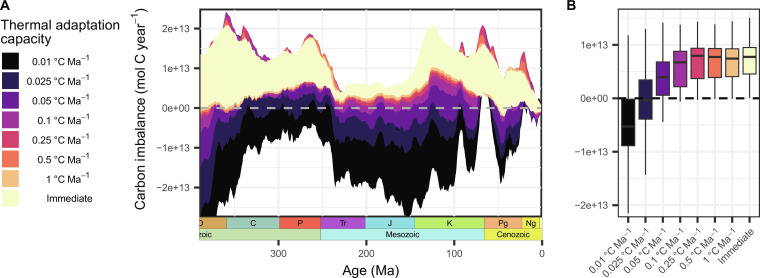
Atmosphere-ocean carbon mass balance for different capacities of thermal adaptation of terrestrial floras. (**A**) Temporal evolution of imbalance. The colored area for each vegetation model implementation represents the uncertainty in the mass balance for different CO_2_/climate and solid Earth degassing reconstructions. The dashed line at 0 depicts the expected mass balance according to the paleothermostat theory. (**B**) Distribution of the median flux imbalances per time step across the model period (0 to 390 Ma at 500-ka time steps). The base dispersal capacity of terrestrial floras is 1300 km Ma^−1^ in all depicted scenarios, except for the immediate scenario, in which floras have an unlimited thermal adaptation and dispersal capacity. Boxes represent the interquartile range, with a line indicating the median. Only data points within a range of 1.5 times the interquartile range are presented (whiskers).

Including eco-evolutionary processes in the reconstruction of the atmosphere-ocean carbon mass balance allows a major reduction in the estimated carbon flux imbalance compared to the systematic surplus with the default configuration with no eco-evolutionary dynamics (Fig. 4). This is especially the case for model implementations that assume the adaptive evolution speed of the climatic niche of terrestrial floras to be slower than the reconstructed average temperature change over the last 390 Ma (∆GAST ≈ 0.2°C Ma^−1^; fig. S2), across variable dispersal capacities (1100 to 1700 km Ma^−1^) (fig. S4). Hereby, it is observed that a larger dispersal and migration capacity is compensatory for a slow speed of in situ thermal adaptation evolution. Overall, except for a period around the Cretaceous-Paleogene boundary, the expected paleothermostat flux balance falls within the modeled uncertainty range of the atmosphere-ocean carbon mass balance given by the explored rates of thermal adaptation, degassing, and climate.

**Fig. 5.**
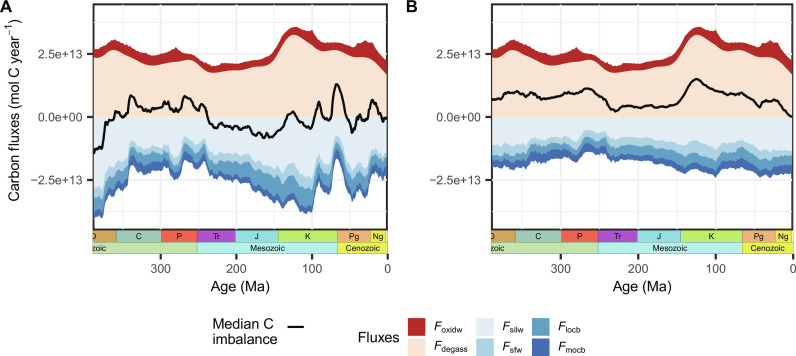
Reconstructed carbon mass balance of the atmosphere-ocean system. Flux estimates represent the median of nine mass balance calculations using different CO_2_ and solid Earth degassing reconstructions over the last 390 Ma, for a vegetation with (**A**) a low capacity to adapt to changes in climate (speed of in situ thermal adaptation evolution of floras of 0.025°C Ma^−1^ and a dispersal range of 1300 km Ma^−1^) and (**B**) an immediate adaptation/no variation in state of adaptation. *F*_oxidw_, oxidative weathering of buried organic carbon; *F*_degass_, total organic and inorganic carbon degassing; *F*_silw_, silicate weathering; *F*_sfw_, seafloor weathering; *F*_locb_, land organic carbon burial; *F*_mocb_, marine organic carbon burial. The single fluxes and their uncertainty ranges are depicted in fig. S5.

Assuming a slow speed in the adaptation to environmental changes results in a closer match of reconstructed carbon source and sink fluxes over time, in better agreement with the paleothermostat theory that suggests a close to steady-state carbon-climate regulation system (Fig. 5). For a vegetation model implementation with floras having thermal adaptation evolution speeds as low as 0.025°C Ma^−1^ and 0.05°C Ma^−1^ (at a dispersal capacity of 1300 km Ma^−1^), mean imbalances over the last 390 Ma of *I* = −1 ± 25% and *I* = 14 ± 20% are modeled, respectively (see fig. S6 for temporal evolution in %). This is a reduction by 25% and 12% of the systematic deviation from an expected long-term flux balance compared to the model with no eco-evolutionary dynamics (*I* = 26%). While the latter model (0.05°C Ma^−1^ adaptation speed) still maintains an overall C source surplus, it is well balanced since the early Mesozoic (fig. S6), indicating the potential importance of variable adaptation capacities through time.

## DISCUSSION

Our analyses support the presence of a climate-driven regulation system that keeps long-term carbon fluxes in balance and thereby maintains habitable conditions on Earth’s surface (*25*–*30*). We further demonstrate that the consideration of eco-evolutionary adaptation dynamics of the terrestrial vegetation results in a major reduction of the systematic deviation of reconstructed carbon fluxes from a long-term atmosphere-ocean carbon mass balance. As such, we argue for the importance of biological climate control mechanisms in altering Earth’s long-term climate.

Current mechanistic understanding of the long-term carbon cycle assumes that terrestrial and marine ecosystems are able to instantaneously keep pace with climatic changes by adapting immediately to the new conditions (*14*, *16*). In contrast, we found that the overall best balanced carbon flux reconstruction over the last 390 Ma is obtained for a vegetation that only slowly adapts to temperature changes due to a limited capacity of floras for in situ thermal adaptation evolution and for dispersal in a given configuration of continental land masses. It should be noted that a close mass balance here only confirms the stability of the carbon cycle as it is currently understood; thus, we consider a large uncertainty in reconstructed abiotic conditions (e.g., CO_2_ and climate) and carbon fluxes (e.g., degassing). When doing so, for most of the considered time period, a flux balance in the global carbon cycle as suggested by the paleothermostat theory is only observed when considering some degree of a limited biotic adaptation capacity, causing an increased variability of organic and inorganic carbon burial that matches reconstructed changes in carbon source fluxes.

The observed effect of eco-evolutionary dynamics on vegetation-related carbon fluxes is robust across different other proposed mechanisms that can affect the atmosphere-ocean carbon mass balance. Tested scenarios include different levels of silicate weathering-climate feedback strengths, different forms of vegetation-weathering feedback, vegetation CO_2_ fertilization effects, and organic carbon burial-erosion feedback (fig. S7). Our results suggest the degree of thermal adaptation and the capacity of the vegetation to respond to climatic changes to be a plausible additional mechanism to explain variation in organic carbon fluxes as well as inorganic carbon burial through the up- or down-regulation of silicate mineral weathering on land, with profound effects on Earth’s carbon mass balance and climate on a million-year timescale.

The rates of thermal adaptation evolution of terrestrial floras considered in the present study (from 0.01 to 1°C Ma^−1^) are in the range of data-based estimates on speeds of climatic niche evolution in plants (*21*). The observed particular importance of low adaptation capacities for balancing the carbon mass balance needs to be considered in the context of a smoothed temperature curve reconstructed from the proxy record (∆GAST ≈ 0.2°C Ma^−1^ over the last 390 Ma), resulting in an underestimation of the abruptness and severity of climatic changes at a higher temporal resolution that would severely impact vegetation functioning even at a higher biotic adaptation capacity (e.g., mass extinctions). Further, with a uniform rate of thermal adaptation evolution and dispersal in space and time as well as a constant plant physiology, vegetation evolution and adaptation in our model is represented in a simplified manner. Climate adaptation of organisms is the result of a variety of ecological and evolutionary processes acting on different spatial and temporal scales (*36*–*40*), with the resulting diversity of terrestrial ecosystems and their specific adaptation mechanisms and interactions with the carbon cycle going beyond the effects on organic carbon burial and silicate weathering captured with the present model. For example, different states of adaptation might translate into different plant types present in a specific location (e.g., deep rooting forest versus opportunistic pioneer species) with characteristic interactions with their environment. Nevertheless, our results emphasize how an improved understanding of the spatiotemporal evolution of such ecosystems and their ecological dynamics and effects on biogeochemical cycles may help to understand past climatic shifts and to resolve remaining discrepancies between simulations and paleoclimate proxies. For example, current modeling is not able to reproduce the degree of warming during the Early Triassic greenhouse (*14*), which may be related to the assumption of geologically instantaneous biotic adaptation, in which plants are assumed to rapidly bury more carbon when temperatures rise. Integrating eco-evolutionary dynamics into deep time Earth system models to account for the disruption and potentially slow response time of the vegetation to environmental change would likely result in more climate warming for smaller inputs of CO_2_ to the atmosphere and suggests a re-evaluation of the inputs of carbon required to cause hyperthermal events.

The present study highlights how ecological and evolutionary dynamics of the vegetation can strongly interfere with the long-term carbon-climate regulation system, affecting both organic and inorganic carbon fluxes. Considering a higher sensitivity of the geologic carbon-climate regulation system to such biological dynamics may be central to advance our understanding of Earth’s past and future climatic evolution.

## MATERIALS AND METHODS

The long-term atmosphere-ocean carbon mass balance can be written as

$$\frac{dM_{\text{ao}}}{\mathrm{dt}}=F_{\text{degass}}+F_{\text{oxidw}}-F_{\text{locb}}-F_{\text{mocb}}-F_{\text{silw}}-F_{\text{sfw}}$$ (1)

with *M*_ao_ being the mass of carbon in the atmosphere-ocean pool, *t* the time, *F*_degass_ the carbon degassing, *F*_oxidw_ the oxidative weathering organic carbon, *F*_locb_ the burial of organic carbon from land, *F*_mocb_ the burial of marine organic carbon, *F*_silw_ the weathering of continental silicate minerals, and *F*_sfw_ the seafloor weathering (*14*). The paleothermostat theory assumes that on timescales of several hundred thousand years, atmospheric CO_2_ stabilizes at a concentration at which carbon removal and release fluxes are in balance. Accordingly, a flux imbalance (*I*) between removal and release can be calculated as

$$I=1-\frac{\left(F_{\text{locb}}+F_{\text{mocb}}+F_{\text{silw}}+F_{\text{sfw}}\right)}{\left(F_{\text{degass}}+F_{\text{oxidw}}\right)}$$ (2)

### Vegetation productivity and adaptation model

Organic carbon production and burial (*F*_locb_) from terrestrial grid cells of the model domain was approximated as follows

$$F_{\text{locb}}=\epsilon^{*}\cdot \text{RSS}\cdot T_{\text{lim}}\cdot A_{\text{lim}}\cdot \Omega$$ (3)

where ϵ^∗^ represents a radiation conversion efficiency that is scaled to obtain the present-day land-derived organic carbon burial rate of approximately 3.5 × 10^12^ mol C year^−1^ (*16*); *RSS* is the surface net shortwave radiation; *T*_lim_ is a temperature limitation factor that is 0 for annual average temperatures lower than −30°C and higher than 45°C, and 1 for temperatures between −20° and 35°C and linearly interpolated for transition temperatures; and *A*_lim_ is an aridity limitation of productivity based on an aridity index $\left(\text{aridity index}=\frac{\text{net surface shortwave radiation}}{\text{latent heat}\cdot \text{precipitation}}\right)$ , normalized to 0 (hyper-arid, *aridity index* >7) and 1 (humid). Finally, for the models that consider eco-evolutionary adaptation dynamics to climatic changes, Ω (between 0 and 1) represents the state of adaptation of the flora that is present in a specific terrestrial grid cell to the local temperature conditions. The state of adaptation is derived using an eco-evolutionary vegetation model that was built based on the gen3sis model framework (*35*). In the model, every terrestrial grid cell is populated by a biological unit that is called the grid cell’s “flora,” characterized by an optimum temperature for productivity (*T*_opt_) (resulting in as many floras as terrestrial grid cells). At the beginning of the simulations (390 Ma ago), the *T*_opt_ values of the terrestrial floras were randomly set equal to the grid cell’s temperature ± 3°C. At a time step of 500 ka, the terrestrial floras can disperse into other terrestrial grid cells within a specified dispersal range (the base dispersal capacity is varied between 1100 and 1700 km Ma^−1^ and scales with local aridity conditions) and occupy the cell if the arriving flora is better adapted to the local climate than the already present flora. In addition to this dispersal-based adaptation to the changing climate, floras can evolve their *T*_opt_ toward the local environment at a specified speed [varied between 0.01 and 1°C Ma^−1^ (*21*)], accounting for in situ thermal adaptation evolution. The state of adaptation Ω of a flora present in a specific terrestrial grid cell is then calculated as a function of the difference between the local temperature (*T*_local_) and the flora’s *T*_opt_ according to

$$\Omega =\text{exp}\left[-k\cdot {\left(T_{\text{opt}}-T_{\text{local}}\right)}^{2}\right]$$ (4)

with *k* being a penalty parameter accounting for the impact of a maladaptation to the local climate. With paleogeographic and climatic reconstructions as inputs of the gen3sis model, the implementation of different speeds of in situ thermal adaptation evolution and dispersal capacities results in a set of eco-evolutionary trajectories, representing a spatially explicit estimation of the vegetation’s state of climate adaptation through time. With all models being calibrated to reproduce present-day reference global carbon fluxes, predictions of higher or lower abundances of well-adapted floras in the geologic past compared to what is modeled for the present day will result in higher or lower global carbon fluxes in the past, respectively.

Changing the penalty parameter *k* in Eq. 4 has a similar effect as changing the biotic adaptation capacity, by increasing or reducing the sensitivity to climatic changes. The effect of changing *k* (default value *k* = 0.25) on the effective modeled sensitivity of primary productivity to temperature changes as well as the atmosphere-ocean carbon mass balance is illustrated in fig. S8.

Marine organic carbon production and burial (*F*_mocb_) is approximated using a high-order polynomial function of sea surface temperatures, empirically derived by Behrenfeld and Falkowski (*41*) and commonly used to describe physiological constrains of phytoplankton productivity in the ocean in chlorophyll-based productivity estimates (*42*). The polynomial accounts for temperature limitation of productivity in low temperature ranges and stratification induced nutrient limitations at high temperatures.

$$\begin{array}{c} F_{\text{mocb}}=\gamma^{*}\cdot (-3.24\cdot 10^{-8}\;\mathrm{SS}T^{7}+3.4132\cdot 10^{-6}\;\text{SS}T^{6}-\\ 1.3248\cdot 10^{-4}\;\text{SS}T^{5}+2.462\cdot 10^{-3}\;\text{SS}T^{4}-0.0205\;\text{SS}T^{3}+\\ 0.0617\;\text{SS}T^{2}+0.2749\;\text{SST}+1.2956) \end{array}$$ (5)

With γ^∗^ being a base rate of marine productivity, scaled to obtain a global *F*_mocb_ flux of approximately 3.5 × 10^12^ mol C year^−1^ using present-day climatic and geographic boundary conditions, and *SST* being the sea surface temperature in the considered ocean grid cell. The assumed present-day sum of organic matter production and burial of terrestrial and marine origin (*F*_locb_ + *F*_mocb_) amounts to 7 × 10^12^ mol C year^−1^. This is in the range of estimates for present-day total organic carbon burial (*16*) and was chosen so that the fraction of organic carbon burial at present day amounts to 0.20 to 0.25% of the modeled total gross carbon burial flux (*43*).

### Abiotic carbon fluxes

The spatially explicit calculations of silicate weathering are based on the formulation by West (*44*), with the silicate cation denudation flux of a grid cell (ω_silw_) being a function of local runoff (*Q*), temperature (*T*), and erosion (ε). Additionally, a spatially explicit feedback of local terrestrial plant productivity on silicate weathering is introduced (*f*_NPP_), which strongly increases weathering in areas of high primary productivity and no mineral supply limitation by erosion, resulting in

$$\omega_{\text{silw}}=\varepsilon \cdot \chi_{m}\cdot \left[1-e^{-K\cdot f\left(Q\right)\cdot f\left(T\right)\cdot f\left(\varepsilon \right)}\right]\cdot f_{\text{NPP}}$$ (6)

Erosion rates are calculated as a function of topographic slope (*s*) and local runoff (*45*), according to

$$\varepsilon =k_{e}\cdot Q_{i}^{0.5}\cdot s$$ (7)

with *k*_e_ representing a scaling parameter to obtain the estimated present-day rate of erosion with present-day topography and runoff rates (global sum of approx. 16 Gt year^−1^). The plant productivity feedback *f*_NPP_ scales local silicate weathering rates with the normalized productivity potential (*NPP*_norm_, 1 no abiotic or eco-evolutionary adaptation limitation of productivity, 0 no plant productivity) and defines the weathering intensity in the absence of plants [derived from (*16*)]:

$$\text{NP}P_{\text{norm}}=\frac{\text{RSS}}{\text{RS}S_{\text{max}}}\cdot T_{\text{lim}}\cdot A_{\text{lim}}\cdot \Omega$$ (8)

$$f_{\text{NPP}}=\left[1-\text{min}\left(\text{NP}P_{\text{norm}},1\right)\right]\cdot \text{PREPLANT}+\text{NP}P_{\text{norm}}$$ (9)

*RSS*_max_ determines a limit for radiation limitation (5000 MJ m^−2^ year^−1^). *PREPLANT* of 0.1 (default) or 0.25 corresponds to a 10-fold or a 4-fold increase of weathering rates in the presence of highly productive floras, respectively. The spatial distribution of primary productivity and weathering enhancement potential for the last model time step (500 ka to present) for different adaptation capacities is presented in fig. S9.

Finally, the global amount of CO_2_ drawdown by silicate weathering is assumed proportional to the total cation denudation flux, assuming a present-day rate of silicate weathering CO_2_ drawdown of *F*_silw_ = 1.325 × 10^13^ mol C year^−1^ (*14*) with present-day topography and climate. A map of the spatial distribution of silicate weathering rates for the present day is shown in fig. S10D.

Global oxidative weathering of organic carbon is calculated as a function of erosion rates and atmospheric oxygen concentrations (*14*, *46*)

$$F_{\text{oxidw}}=k_{\text{oxidw}}\cdot \left(\frac{\varepsilon_{t}}{\varepsilon_{0}}\right)\cdot {\left(\frac{O_{2,t}}{O_{2,0}}\right)}^{0.5}$$ (10)

with *k*_oxidw_ representing the present-day rate of oxidative weathering (assuming a flux balance for the present day following Eq. 4: 5.75 × 10^12^ mol C year^−1^), ε_0_ the present-day erosion rate, ε*_t_* the erosion rate at time *t*, O_2,0_ the present-day atmospheric oxygen concentration, and *O*_2,*t*_ the oxygen concentration at time *t*, derived from the reconstruction by Glasspool and Scott (*32*).

Seafloor weathering was calculated following (*14*)

$$F_{\text{sfw}}=k_{\text{sfw}}\cdot e^{0.0608\left(T_{\text{GAST},t}-T_{\text{GAST},0}\right)}\cdot \frac{D_{t}}{D_{0}}$$ (11)

with *k*_sfw_ representing the present-day rate of seafloor weathering rate (approximately 1.75 × 10^12^ mol C year^−1^), *T*_GAST,*t*_ the global average surface temperature at *t*, *T*_GAST,0_ the present-day global average surface temperature, *D_t_* the normalized degassing rate at *t*, and *D*_0_ the present-day normalized degassing rate.

### Degassing

The carbon balance calculations were conducted considering four different estimates on the temporal evolution of global degassing rates during the last 390 Ma that are also used in other Phanerozoic biogeochemical models (fig. S5) (*14*, *47*). The higher limit in degassing is derived from kinematic plate modeling of total material destruction rates (*48*, *49*). The lower limit of the degassing curve originates from estimates on subduction zone lengths and continental rifting (*50*, *51*). Then, an intermediate scenario between these two was considered (*14*). Finally, an additional degassing estimate by Marcilly *et al.* (*47*) was considered for periods during which their estimate was lower than the uncertainty range given by the other methods. The latter represents an estimate based on the age-frequency distribution of arc-associated detrital zircons. Present-day rates of degassing were taken to be 1.25 × 10^12^ mol C year^−1^ and 15 × 10^12^ mol C year^−1^ for organic and inorganic carbon degassing, respectively (*16*). For the inorganic carbon degassing, it was further assumed that the evolution of pelagic calcifiers shifted the export of carbonate to the deep sea and, as deep-sea sediment is more likely to be subducted compared to sediment deposited on shelves, increased inorganic carbon degassing. This was accounted for by multiplying inorganic carbon degassing with a scaling factor that amounts to 0.75 before 150 Ma and which is linearly increasing to its present-day value of 1 from 150 Ma to 100 Ma before present (*14*).

### Climate and topography

Topographic information for the flux calculations as well as for the climate modeling were taken from Scotese and Wright (*31*), spatially interpolated to a 3.75° × 3.75° resolution and temporally to a 500-ka resolution. To obtain climatic information on temperature, aridity, radiation, and runoff for the flux calculations, the Earth system model of intermediate complexity PlaSim (version 17), coupled to a mixed-layer slab ocean, was used (*34*). The model was run for 150 model years, and the average of the last 50 model years was used for the carbon flux calculations. Further, the model was run for a uniform surface albedo on land (0.2) as well as a constant soil depth (0.15 m). Temperatures, radiation, and aridity modeled for the present-day geography and pre-industrial CO_2_ levels are shown in fig. S10. By running PlaSim in 5-Ma intervals from 390 Ma ago to the present and for seven CO_2_ concentrations between 50 and 3000 ppm for each time interval, a climate lookup table was created, which allowed an approximation of climatic conditions for every 500-ka time step of the flux calculations as well as for different CO_2_ trajectories. We considered three CO_2_ trajectories over the last 390 Ma, identical to the median and the lower and upper bound of the 68% confidence interval of atmospheric CO_2_ concentrations reported by Foster *et al.* (*33*) (fig. S2). The median difference between the lower and upper considered CO_2_ concentration for each carbon flux calculation time step amounts to 490 ppm, covering a wide range of uncertainty regarding the surface climatic condition for each time step due to uncertainty in atmospheric greenhouse gas concentrations or the activity of other natural climate drivers (e.g., solar or volcanic activity). Combined, the flux calculations for each parametrization of the vegetation adaptation capacity (defined by dispersal range and thermal evolution rate) were conducted for nine combinations of degassing and climate evolution over the last 390 Ma.

### Illustration of imbalance effect on atmospheric CO_2_

To illustrate the potential effect of a carbon mass imbalance on atmospheric CO_2_, we apply the model by Kump and Arthur (*52*), assuming atmospheric CO_2_ concentrations to be proportional to the square of change in the total mass of carbon in the atmosphere-ocean pool

$${\text{CO}}_{2}={\left(\frac{M_{\text{ao}}}{M_{\text{ao},\text{ref}}}\right)}^{2}\cdot {\text{CO}}_{2,\text{ref}}$$ (12)

with *M*_ao,ref_ and CO_2,ref_ being the CO_2_ concentration and the corresponding atmosphere-ocean carbon content suggested for pre-industrial time [following Berner and Caldeira (*25*): *M*_ao,ref_ = 3 × 10^18^ mol C and CO_2,ref_ = 280 ppm]. The model assumes a constant mass of oceanic Ca^2+^ and an overall ocean saturation with respect to CaCO_3_.

### Additional sensitivity tests

In addition to the mentioned uncertainties in the eco-evolutionary adaptation capacity of the vegetation (dispersal and speed of thermal adaptation), carbon degassing, and climate considered in the estimation of past carbon fluxes, the effect of several alternative mechanisms with potential effects on the atmosphere-ocean carbon mass balance was tested and is reported in the Supplementary Materials. These include a feedback of physical erosion on terrestrial organic carbon burial as proposed by Hilton (*53*). Hereby, the rate of total organic carbon burial was assumed to linearly scale with the average global erosion rate by a factor of 1 or 4. Further, different sensitivities of the weathering formulation of West (*44*) (Eq. 6) to temperature and runoff as proposed by Penman *et al.* (*54*) were considered by varying the activation energy of silicate weathering between 10 and 40 kJ mol^−1^ and by varying the runoff dependency *k_w_* between 1.5 × 10^−6^ mm year^−1^ and 1 × 10^−3^ mm year^−1^ [for more details on the weathering parameters, see West (*44*) and Maffre *et al.* (*45*)]. Further, by increasing the *PREPLANT* parameter (Eq. 9) from 0.1 to 0.25, the effect of a reduced effect of plant primary productivity on silicate weathering rates was assessed (4-fold instead of 10-fold weathering increase in highly productive areas). Then, the possibility of a CO_2_ fertilization effect on plant productivity and thus weathering enhancement was considered by extending the primary productivity function (Eq. 3) by a fertilization function as used in the GEOCARB model suite $\left[f\left({\mathrm{CO}}_{2}\right)={\frac{\left(2\cdot \text{RC}O_{2}\right)}{\left(1+\text{RC}O_{2}\right)}}^{0.4}\right]$. Finally, also following GEOCARB, the possibility of a strong CO_2_ dependency of weathering in the absence of plants is considered by replacing *PREPLANT* in Eq. 9 with $\left(\text{PREPLANT}\cdot \text{RC}O_{2}^{0.5}\right)$.

## Supplementary Materials

### This PDF file includes:

Figs. S1 to S10 References
