# Supplementary material for: Speed of thermal adaptation of terrestrial vegetation alters Earth’s long-term climate
Source: Sci Adv. 2024 Mar 1;10(9):eadj4408. doi: 10.1126/sciadv.adj4408 (PMC10906918; doi:10.1126/sciadv.adj4408)
Supplement: Supplementary file 1 — Figs. S1 to S10 References [file sciadv.adj4408_sm.pdf]

Science Advances
AAAS

Supplementary Materials for

Speed of thermal adaptation of terrestrial vegetation alters Earth's long-term climate

Julian Rogger *et al.*

Corresponding author: Julian Rogger, julian.rogger@erdw.ethz.ch

Sci. Adv. **10**, eadj4408 (2024)
DOI: 10.1126/sciadv.adj4408

This PDF file includes:

Figs. S1 to S10
References

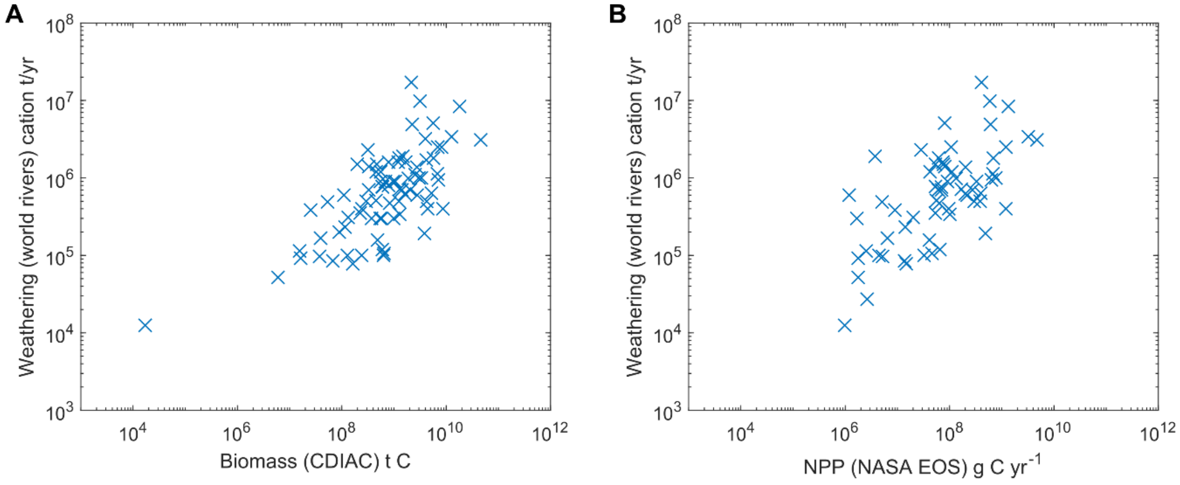

Figure S1: Vegetation-weathering relationship. Relationship between catchment scale silicate weathering and A) catchment standing biomass, B) catchment net primary productivity rates. Catchment maps from (*45*), weathering fluxes from (*55*), biomass and net primary productivity (NPP) data from CDIAC and NASA EOS data bases, respectively.

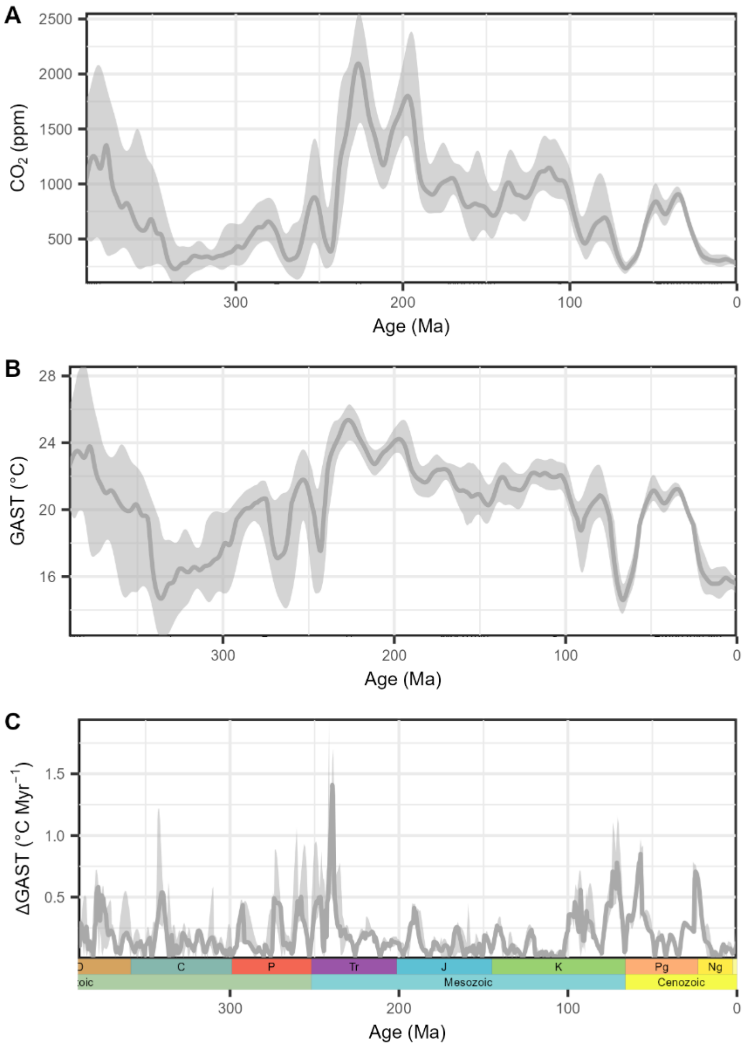

Figure S2: Input data for CO_2 and predicted temperatures. A) Range of atmospheric CO_2 considered, based on reconstruction by Foster *et al.* (*33*), B) predicted global average surface temperature (GAST) using the CO_2 trajectories and the intermediate complexity climate model PlaSim (*34*). The obtained temperature curve agrees with the major temperature trends obtained for the Phanerozoic using isotope data (e.g., by Scotese *et al.* (*56*)), but differs in the timing and magnitude of fluctuations and temperature extremes. C) Absolute temperature changes between model time steps.

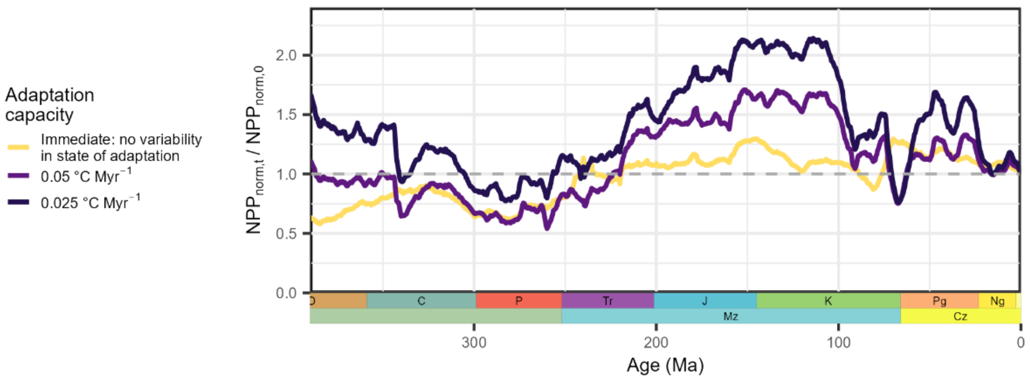

Figure S3: Temporal evolution of area-weighted global average NPP_{norm}. The normalized primary productivity and weathering potential NPP_{norm} (eq. 8) is a measure of how abiotic environmental conditions (temperature, aridity, radiation), the vegetation's adaptation state, and total productive land area affect the global fluxes of organic and inorganic carbon burial (through f_{NPP}, eq. 9). As the model is calibrated to reproduce present-day levels of global organic and inorganic carbon burial, the ratio of NPP_{norm} at time t to NPP_{norm} modelled for the present day ($t = 0$) drives the temporal evolution of the calculated fluxes. As no variability in the state of adaptation occurs in the 'immediate adaptation' scenario, its trajectory represents changes in biotic effects solely due to changes in abiotic environmental conditions. Trajectories for only one of three climatic reconstructions are shown.

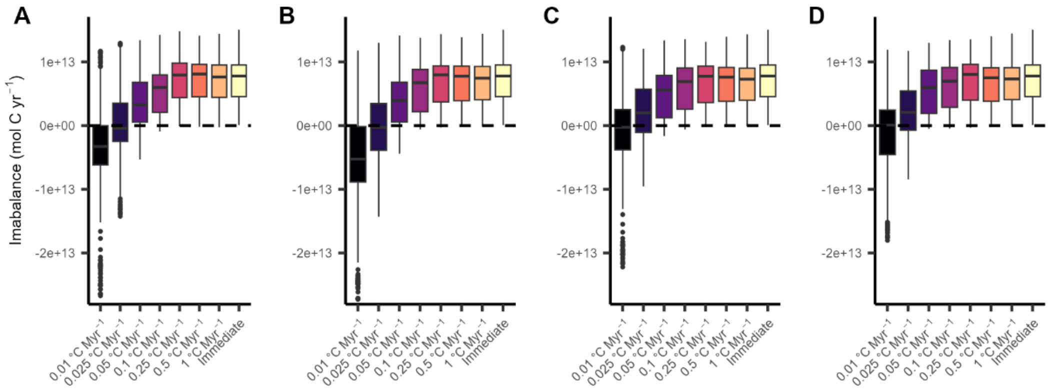

Figure S4: Atmosphere-ocean carbon mass imbalance over the last 390 Myr for different dispersal capacities and speeds of thermal adaptation evolution. A) flora dispersal capacity of 1100 km Myr^{-1}, B) 1300 km Myr^{-1}, C) 1500 km Myr^{-1}, D) 1700 km Myr^{-1}. The horizontal dashed line depicts the expected carbon mass balance according to the paleothermostat theory.

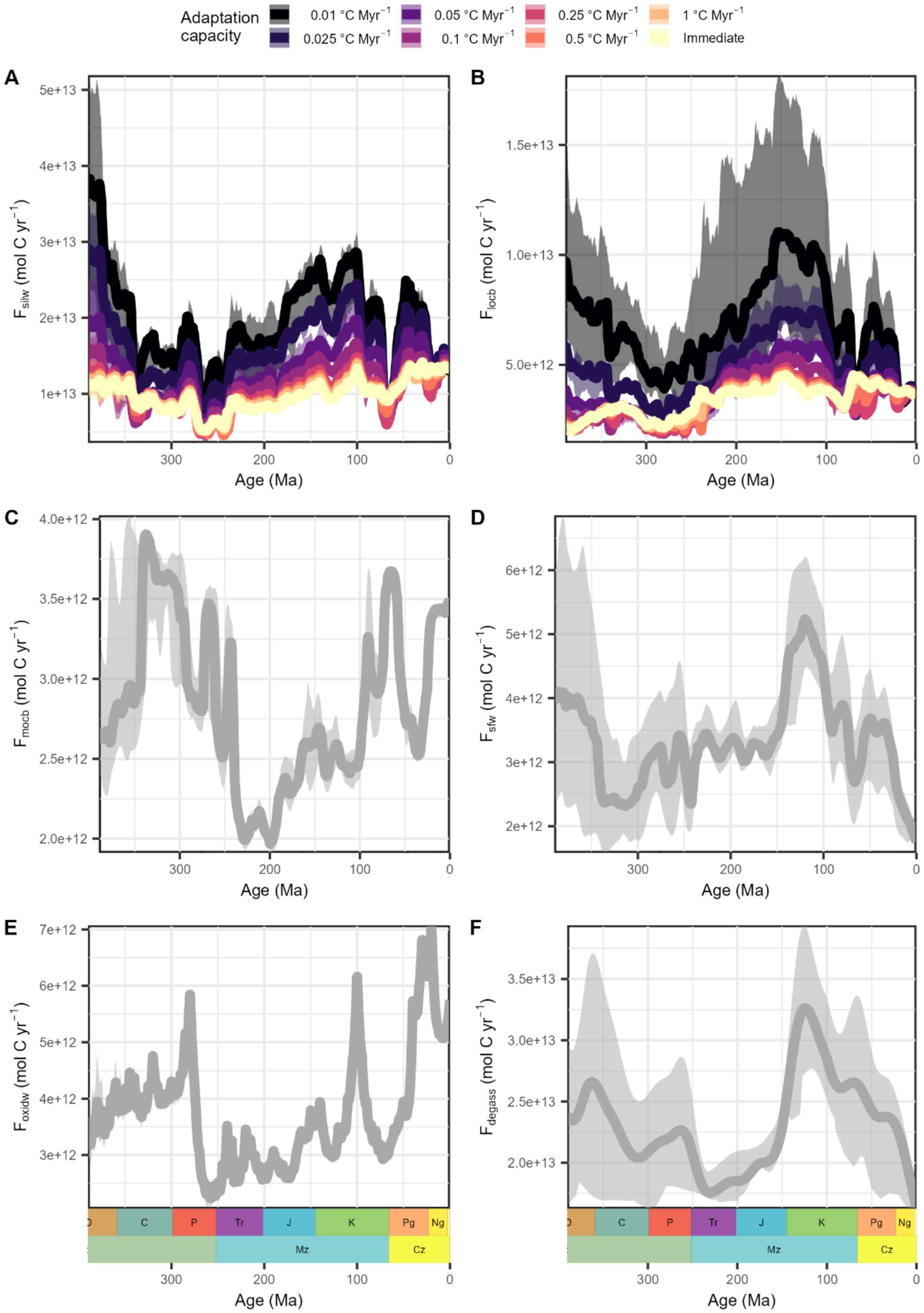

Figure S5: Reconstructed carbon fluxes. A) Carbon consumption by silicate weathering (F_{silw}) for different vegetation adaptation capacities, B) terrestrial organic carbon burial (F_{locb}) for different vegetation adaptation capacities (A and B for constant dispersal capacity of 1300 km Myr^{-1}), C) marine organic carbon burial (F_{mocb}), D) seafloor weathering (F_{sfw}), E) oxidative weathering of buried organic carbon (F_{oxidw}), F) solid Earth degassing of organic and inorganic carbon (F_{degass}).

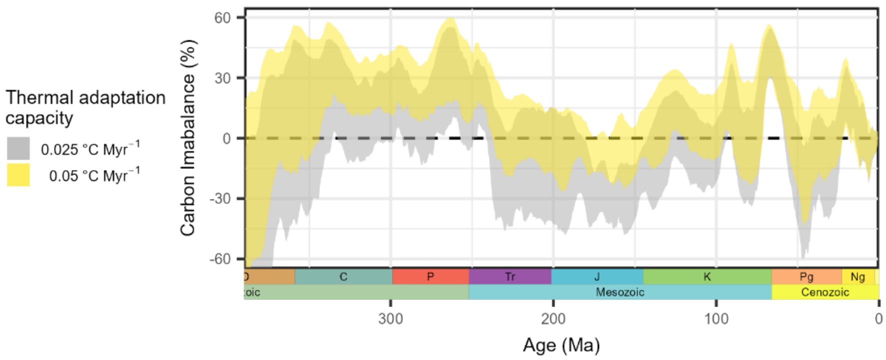

Figure S6: Atmosphere-ocean carbon imbalances reconstructed for two vegetation implementations considering eco-evolutionary adaptation dynamics. The colored area represents the uncertainty in the mass balance for different CO_2/climate and solid Earth degassing reconstructions. The dashed line at 0% depicts the expected mass balance according to the paleothermostat theory.

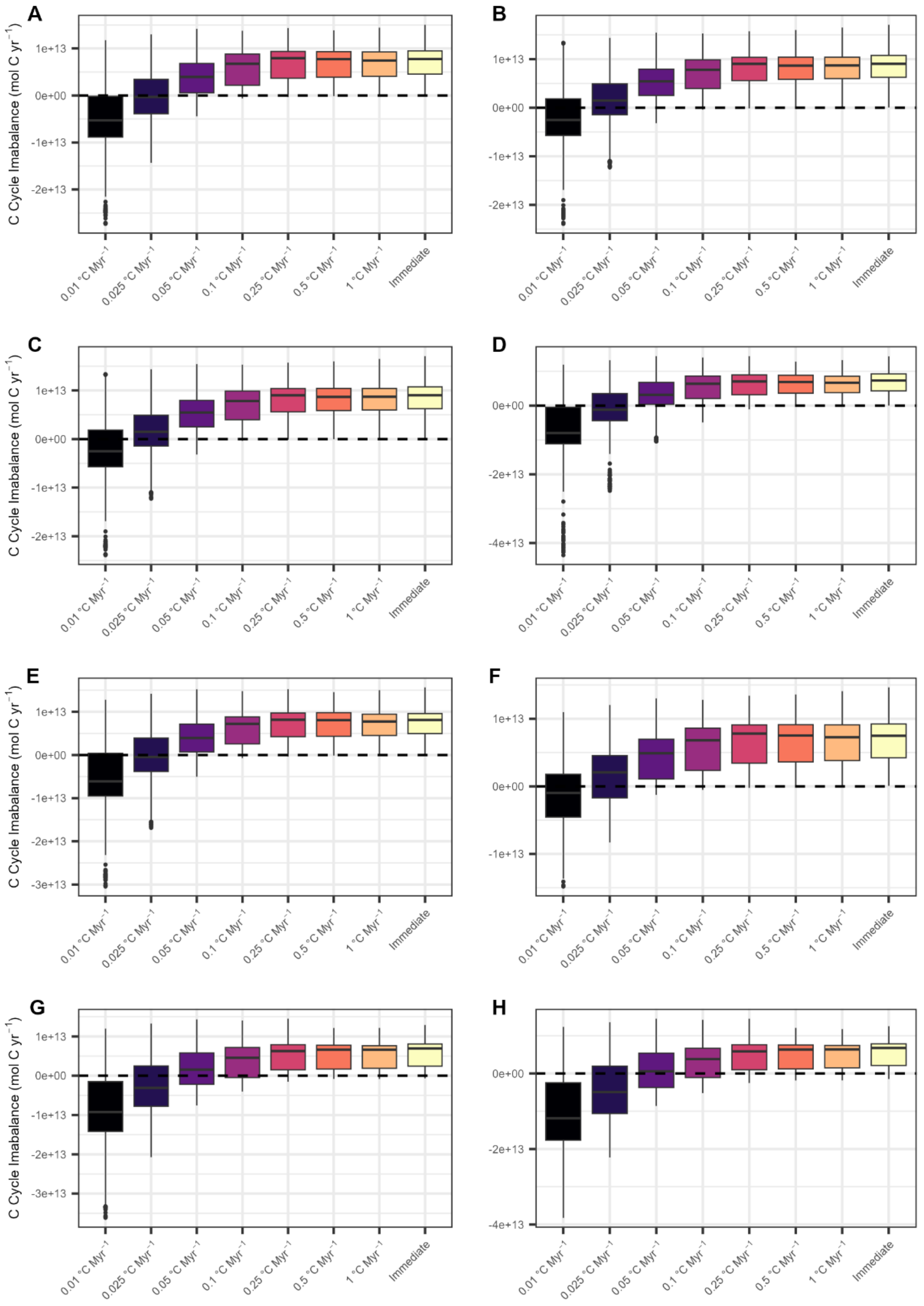

Figure S7: Distribution of flux imbalances over last 390 Myr considering alternative mechanisms that affect the long-term atmosphere-ocean carbon mass balance. A) Reference model, B) linear scaling of land organic carbon burial (F_{locb}) with global erosion rates as proposed by Hilton (*53*) (1% increase in erosion = 1% increase in F_{locb}), C) stronger F_{locb}-erosion feedback (1% increase in erosion = 4% increase in F_{locb}), D) different responsiveness of silicate weathering to climate changes (temperature, runoff) as proposed by Penman *et al.* (*54*): low activation energy of silicate weathering reaction (10 kJ mol^{-1}), weak runoff dependency (k_w = 1.5e^{-6} mm yr^{-1}), E) high silicate weathering activation energy (40 kJ mol^{-1}) and strong runoff dependency (k_w = 1e^{-3} mm yr^{-1}). For more details on the weathering reaction

parameters, see West (*44*) and Maffre *et al.* (*45*). F) Reduced maximum enhancement effect of plant productivity on silicate weathering rates (maximum = 4-fold, compared to 10-fold in reference model), G) consideration of a strong CO_2 fertilization effect on plant productivity and thus, indirectly on silicate weathering rates; implemented following the GEOCARB model suite (*15*), H) consideration of a strong CO_2 driven weathering feedback in the absence of plants, also following the GEOCARB model (*15*). Boxes represent the interquartile range, with a line indicating the median. Whiskers include data points within 1.5 times the interquartile range.

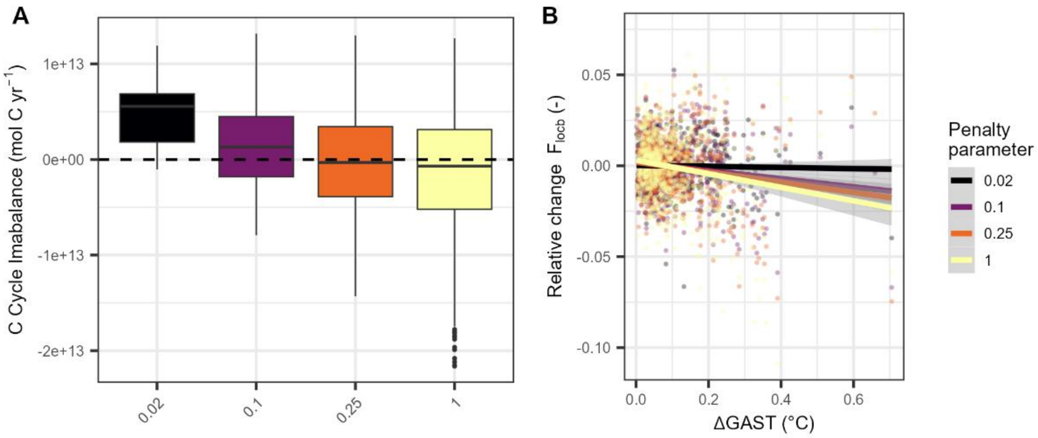

Figure S8: Sensitivity to penalty parameter *k*. Effect on A) carbon mass balance and B) terrestrial derived organic matter burial. Default value considered in the study is k =0.25, with larger numbers representing a higher sensitivity of terrestrial floras to temperature changes. Lines indicate a linear fit for each penalty parameter.

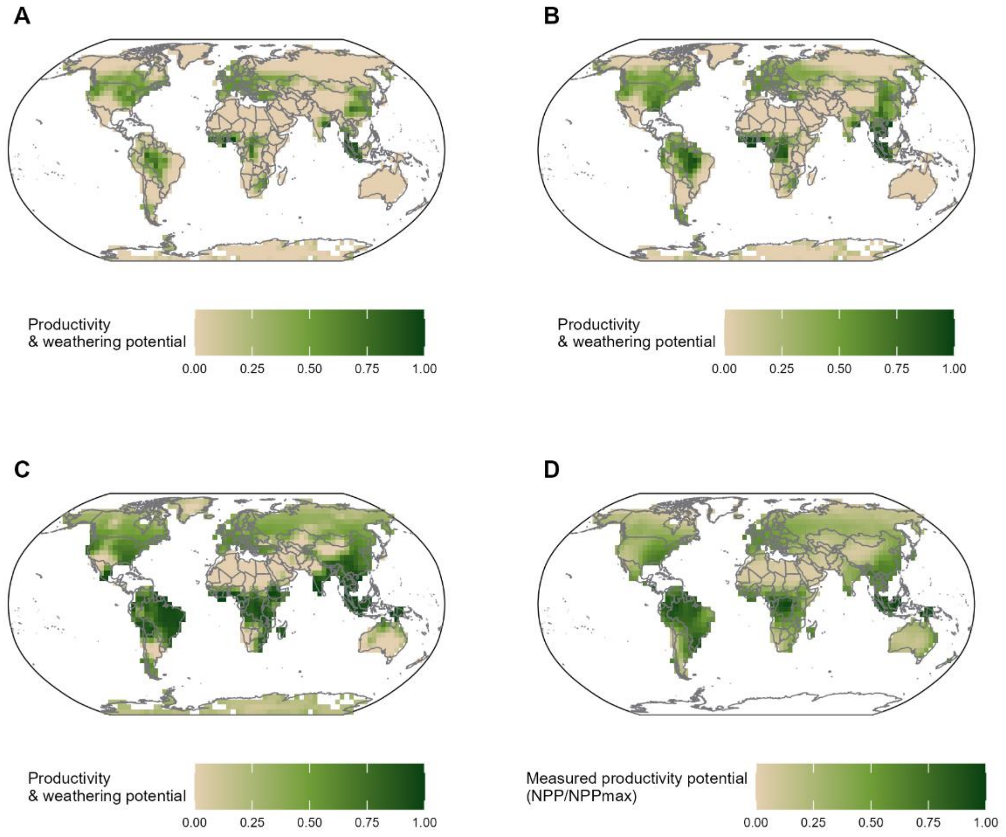

Figure S9: Normalized primary productivity potential (NPP_{norm}; eq. 8) for last model time step (500 kyr to present) for different vegetation model implementations. A) 0.025°C Myr^{-1} thermal adaptation evolution speed, B) 0.05°C Myr^{-1}, C) immediate adaptation model (no eco-evolutionary dynamics). CO_2 of last model time step amounts to ~276 ppm. D) semi-empirical (satellite data + modelled) net primary productivity (NPP) data for the present day (1931-1961, 340-360 ppm CO_2), normalized to a 0-1 range for comparison (*57*).

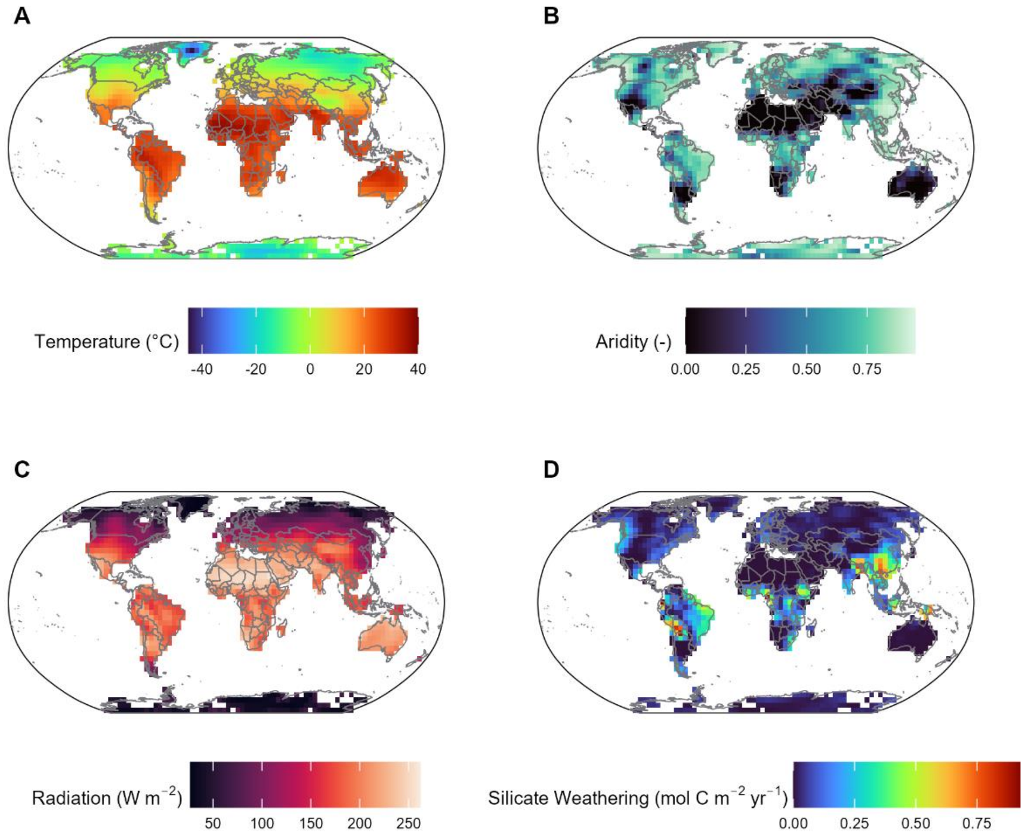

Figure S10: Climate data and silicate weathering distribution for present day. A) Modelled surface temperatures for pre-industrial CO_2 levels using the PlaSim climate model (*34*), B) normalized aridity index, C) surface net shortwave radiation, D) estimated rates of silicate weathering carbon consumption for an immediately adapting vegetation model.

REFERENCES AND NOTES

1. R. Berner, A model for atmospheric CO_2 over Phanerozoic time. *Am. J. Sci.* **291**, 339–376 (1991).

2. T. W. Dahl, S. K. Arens, The impacts of land plant evolution on Earth's climate and oxygenation state—An interdisciplinary review. *Chem. Geol.* **547**, 119665 (2020).

3. L. L. Taylor, J. R. Leake, J. Quirk, K. Hardy, S. A. Banwart, D. J. Beerling, Biological weathering and the long-term carbon cycle: Integrating mycorrhizal evolution and function into the current paradigm. *Geobiology* **7**, 171–191 (2009).

4. E. Berner, R. Berner, K. Moulton, Plants and mineral weathering: Present and past, in *Treatise on Geochemistry*, K. K. Turekian, H. D. Holland (Elsevier, 2003), vol. 5, pp. 169–188.

5. K. L. Moulton, Solute flux and mineral mass balance approaches to the quantification of plant effects on silicate weathering. *Am. J. Sci.* **300**, 539–570 (2000).

6. J. Quirk, M. Y. Andrews, J. R. Leake, S. A. Banwart, D. J. Beerling, Ectomycorrhizal fungi and past high CO_2 atmospheres enhance mineral weathering through increased below-ground carbon-energy fluxes. *Biol. Lett.* **10**, 20140375 (2014).

7. J. Quirk, J. R. Leake, D. A. Johnson, L. L. Taylor, L. Saccone, D. J. Beerling, Constraining the role of early land plants in Palaeozoic weathering and global cooling. *Proc. R. Soc. B Biol. Sci.* **282**, 20151115 (2015).

8. L. L. Taylor, S. A. Banwart, P. J. Valdes, J. R. Leake, D. J. Beerling, Evaluating the effects of terrestrial ecosystems, climate and carbon dioxide on weathering over geological time: A global-scale process-based approach. *Philos. Trans. R. Soc. B Biol. Sci.* **367**, 565–582 (2012).

9. P. Porada, B. Weber, W. Elbert, U. Pöschl, A. Kleidon, Estimating impacts of lichens and bryophytes on global biogeochemical cycles. *Global Biogeochem. Cycles* **28**, 71–85 (2014).

10. T. M. Lenton, M. Crouch, M. Johnson, N. Pires, L. Dolan, First plants cooled the Ordovician. *Nat. Geosci.* **5**, 86–89 (2012).

11. J. L. Morris, J. R. Leake, W. E. Stein, C. M. Berry, J. E. A. Marshall, C. H. Wellman, J. A. Milton, S. Hillier, F. Mannolini, J. Quirk, D. J. Beerling, Investigating Devonian trees as geo-engineers of past climates: Linking palaeosols to palaeobotany and experimental geobiology. *Palaeontology* **58**, 787–801 (2015).

12. R. A. Berner, Paleozoic atmospheric CO_2: Importance of solar radiation and plant evolution. *Science* **261**, 68–70 (1993).

13. D. W. Schwartzman, T. Volk, Biotic enhancement of weathering and the habitability of Earth. *Nature* **340**, 457–460 (1989).

14. B. J. Mills, Y. Donnadieu, Y. Goddéris, Spatial continuous integration of Phanerozoic global biogeochemistry and climate. *Gondw. Res.* **100**, 73–86 (2021).

15. R. A. Berner, GEOCARB III: A revised model of atmospheric CO_2 over Phanerozoic time. *Am. J. Sci.* **301**, 182–204 (2001).

16. T. M. Lenton, COPSE reloaded: An improved model of biogeochemical cycling over Phanerozoic time. *Earth Sci. Rev.* **178**, 1–28 (2018).

17. D. Nogués-Bravo, F. Rodríguez-Sánchez, L. Orsini, E. de Boer, R. Jansson, H. Morlon, D. A. Fordham, S. T. Jackson, Cracking the code of biodiversity responses to past climate change. *Trends Ecol. Evol.* **33**, 765–776 (2018).

18. R. T. Corlett, D. A. Westcott, Will plant movements keep up with climate change? *Trends Ecol. Evol.* **28**, 482–488 (2013).

19. L. T. Lancaster, A. M. Humphreys, Global variation in the thermal tolerances of plants. *Proc. Natl. Acad. Sci. U.S.A.* **117**, 13580–13587 (2020).

20. M. Huang, S. Piao, P. Ciais, J. Peñuelas, X. Wang, T. F. Keenan, S. Peng, J. A. Berry, K. Wang, J. Mao, R. Alkama, A. Cescatti, M. Cuntz, H. De Deurwaerder, M. Gao, Y. He, Y. Liu, Y. Luo, R. B. Myneni, S. Niu, X. Shi, W. Yuan, H. Verbeeck, T. Wang, J. Wu, I. A. Janssens, Air

temperature optima of vegetation productivity across global biomes. *Nat. Ecol. Evol.* **3**, 772–779 (2019).

21. H. Liu, Q. Ye, J. J. Wiens, Climatic-niche evolution follows similar rules in plants and animals. *Nat. Ecol. Evol.* **4**, 753–763 (2020).

22. J. M. Bennett, J. Sunday, P. Calosi, F. Villalobos, B. Martínez, R. Molina-Venegas, M. B. Araújo, A. C. Algar, S. Clusella-Trullas, B. A. Hawkins, S. A. Keith, I. Kühn, C. Rahbek, L. Rodríguez, A. Singer, I. Morales-Castilla, M. Á. Olalla-Tárraga, The evolution of critical thermal limits of life on Earth. *Nat. Commun.* **12**, 1198 (2021).

23. C. V. Looy, R. J. Twitchett, D. L. Dilcher, J. H. A. Van Konijnenburg-Van Cittert, H. Visscher, Life in the end-Permian dead zone. *Proc. Natl. Acad. Sci. U.S.A.* **98**, 7879–7883 (2001).

24. Z.-Q. Chen, M. J. Benton, The timing and pattern of biotic recovery following the end-Permian mass extinction. *Nat. Geosci.* **5**, 375–383 (2012).

25. R. A. Berner, K. Caldeira, The need for mass balance and feedback in the geochemical carbon cycle. *Geology*, **25**, 955 (1997).

26. M. P. D'Antonio, D. E. Ibarra, C. K. Boyce, Land plant evolution decreased, rather than increased, weathering rates. *Geology* **48**, 29–33 (2020).

27. E. T. Sundquist, Steady- and non-steady-state carbonate-silicate controls on atmospheric CO_2. *Quat. Sci. Rev.* **10**, 283–296 (1991).

28. J. C. G. Walker, P. B. Hays, J. F. Kasting, A negative feedback mechanism for the long-term stabilization of Earth's surface temperature. *J. Geophys. Res.* **86**, 9776–9782 (1981).

29. R. E. Zeebe, K. Caldeira, Close mass balance of long-term carbon fluxes from ice-core CO_2 and ocean chemistry records. *Nat. Geosci.* **1**, 312–315 (2008).

30. J. K. Caves, A. B. Jost, K. V. Lau, K. Maher, Cenozoic carbon cycle imbalances and a variable weathering feedback. *Earth Planet. Sci. Lett.* **450**, 152–163 (2016).

31. C. Scotese, N. Wright, PALEOMAP Paleodigital Elevation Models (PaleoDEMS) for the Phanerozoic (2018); https://www.earthbyte.org/paleodem-resource-scotese-and-wright-2018.

32. I. J. Glasspool, A. C. Scott, Phanerozoic concentrations of atmospheric oxygen reconstructed from sedimentary charcoal. *Nat. Geosci.* **3**, 627–630 (2010).

33. G. L. Foster, D. L. Royer, D. J. Lunt, Future climate forcing potentially without precedent in the last 420 million years. *Nat. Commun.* **8**, 14845 (2017).

34. K. Fraedrich, A suite of user-friendly global climate models: Hysteresis experiments. *Eur. Phys. J. Plus* **127**, 53 (2012).

35. O. Hagen, B. Flück, F. Fopp, J. S. Cabral, F. Hartig, M. Pontarp, T. F. Rangel, L. Pellissier, gen3sis: A general engine for eco-evolutionary simulations of the processes that shape Earth's biodiversity. *PLOS Biol.* **19**, e3001340 (2021).

36. S. Niu, Y. Luo, S. Fei, W. Yuan, D. Schimel, B. E. Law, C. Ammann, M. Altaf Arain, A. Arneth, M. Aubinet, A. Barr, J. Beringer, C. Bernhofer, T. Andrew Black, N. Buchmann, A. Cescatti, J. Chen, K. J. Davis, E. Dellwik, A. R. Desai, S. Etzold, L. Francois, D. Gianelle, B. Gielen, A. Goldstein, M. Groenendijk, L. Gu, N. Hanan, C. Helfter, T. Hirano, D. Y. Hollinger, M. B. Jones, G. Kiely, T. E. Kolb, W. L. Kutsch, P. Lafleur, D. M. Lawrence, L. Li, A. Lindroth, M. Litvak, D. Loustau, M. Lund, M. Marek, T. A. Martin, G. Matteucci, M. Migliavacca, L. Montagnani, E. Moors, J. William Munger, A. Noormets, W. Oechel, J. Olejnik, K. T. Paw U, K. Pilegaard, S. Rambal, A. Raschi, R. L. Scott, G. Seufert, D. Spano, P. Stoy, M. A. Sutton, A. Varlagin, T. Vesala, E. Weng, G. Wohlfahrt, B. Yang, Z. Zhang, X. Zhou, Thermal optimality of net ecosystem exchange of carbon dioxide and underlying mechanisms. *New Phytol.* **194**, 775–783 (2012).

37. W. Ludwig, S. Hayes, J. Trenner, C. Delker, M. Quint, On the evolution of plant thermomorphogenesis. *J. Exp. Bot.*, erab310 (2021).

38. L. B. Buckley, J. G. Kingsolver, Evolution of thermal sensitivity in changing and variable climates. *Annu. Rev. Ecol. Evol. Syst.* **52**, 563–586 (2021).

39. S. N. Aitken, S. Yeaman, J. A. Holliday, T. Wang, S. Curtis-McLane, Adaptation, migration or extirpation: Climate change outcomes for tree populations. *Evol. Appl.* **1**, 95–111 (2008).

40. M. J. Donoghue, E. J. Edwards, Biome shifts and niche evolution in plants. *Annu. Rev. Ecol. Evol. Syst.* **45**, 547–572 (2014).

41. M. J. Behrenfeld, P. G. Falkowski, Photosynthetic rates derived from satellite-based chlorophyll concentration. *Limnol. Oceanogr.* **42**, 1–20 (1997).

42. M. J. Behrenfeld, R. T. O'Malley, D. A. Siegel, C. R. McClain, J. L. Sarmiento, G. C. Feldman, A. J. Milligan, P. G. Falkowski, R. M. Letelier, E. S. Boss, Climate-driven trends in contemporary ocean productivity. *Nature* **444**, 752–755 (2006).

43. G. A. Shields, B. J. W. Mills, Tectonic controls on the long-term carbon isotope mass balance. *Proc. Natl. Acad. Sci. U.S.A.* **114**, 4318–4323 (2017).

44. A. J. West, Thickness of the chemical weathering zone and implications for erosional and climatic drivers of weathering and for carbon-cycle feedbacks. *Geology* **40**, 811–814 (2012).

45. P. Maffre, J.-B. Ladant, J.-S. Moquet, S. Carretier, D. Labat, Y. Goddéris, Mountain ranges, climate and weathering. Do orogens strengthen or weaken the silicate weathering carbon sink? *Earth Planet. Sci. Lett.* **493**, 174–185 (2018).

46. R. G. Hilton, A. J. West, Mountains, erosion and the carbon cycle. *Nat. Rev. Earth Environ.* **1**, 284–299 (2020).

47. C. M. Marcilly, T. H. Torsvik, M. Domeier, D. L. Royer, New paleogeographic and degassing parameters for long-term carbon cycle models. *Gondw. Res.* **97**, 176–203 (2021).

48. M. Seton, R. Müller, S. Zahirovic, C. Gaina, T. Torsvik, G. Shephard, A. Talsma, M. Gurnis, M. Turner, S. Maus, M. Chandler, Global continental and ocean basin reconstructions since 200Ma. *Earth Sci. Rev.* **113**, 212–270 (2012).

49. M. Domeier, T. H. Torsvik, Full-plate modelling in pre-Jurassic time. *Geol. Mag.* **156**, 261–280 (2019).

50. B. J. Mills, A. J. Krause, C. R. Scotese, D. J. Hill, G. A. Shields, T. M. Lenton, Modelling the long-term carbon cycle, atmospheric CO_2, and Earth surface temperature from late Neoproterozoic to present day. *Gondw. Res.* **67**, 172–186 (2019).

51. S. Brune, S. E. Williams, R. D. Müller, Potential links between continental rifting, CO_2 degassing and climate change through time. *Nat. Geosci.* **10**, 941–946 (2017).

52. L. R. Kump, M. A. Arthur, Interpreting carbon-isotope excursions: Carbonates and organic matter. *Chem. Geol.* **161**, 181–198 (1999).

53. R. G. Hilton, Climate regulates the erosional carbon export from the terrestrial biosphere. *Geomorphology* **277**, 118–132 (2017).

54. D. E. Penman, J. K. Caves Rugenstein, D. E. Ibarra, M. J. Winnick, Silicate weathering as a feedback and forcing in Earth's climate and carbon cycle. *Earth Sci. Rev.* **209**, 103298 (2020).

55. B. J. W. Mills, S. Tennenbaum, D. Schwartzman, Exploring multiple steady states in Earth's long-term carbon cycle. *Am. J. Sci.* **321**, 1033–1044 (2021).

56. C. R. Scotese, H. Song, B. J. Mills, D. G. van der Meer, Phanerozoic paleotemperatures: The earth's changing climate during the last 540 million years. *Earth Sci. Rev.* **215**, 103503 (2021).

57. W. Cramer, D. W. Kicklighter, A. Bondeau, B. M. Iii, G. Churkina, B. Nemry, A. Ruimy, A. L. Schloss, Comparing global models of terrestrial net primary productivity (NPP): Overview and key results. *Glob. Chang. Biol.* **5**, 1–15 (1999).
